# Too Much Information, Too Little Power: The Persistence of Asymmetries in Doctor-Patient Relationships

**DOI:** 10.1080/19428200.2020.1826178

**Published:** 2020-12-03

**Authors:** Cinzia Greco

## Medical Knowledge in the Time of the Internet

Years ago, during an appointment with a breast surgeon in Southern Italy, while I was trying to explain my symptoms, he interrupted me with a wave of the hand and said, “That’s not relevant,” then he dropped a few obscure medical terms with no intention of clarifying them. I left the office with an ambiguous message—Don’t worry, but a surgical intervention is needed—and not much knowledge of what my problem was. Once at home, I did what every patient in the early 2000s was told not to do: I went online and searched for my symptoms and the medical terms that I could remember from the consultation. A few minutes of searching gave me more information than the costly appointment with the medical professional.

In my experience as a young patient, I extensively used medical websites, patient forums and popular medicine articles because I believed that the internet could help me to navigate the difficult relations with the medical establishment. Years later, the problematic nature of medical encounters would emerge as a major factor in the research I conducted exploring the experiences of breast cancer patients in Italy, France and the United Kingdom.^1^ These experiences inform the considerations in the following pages. In short, despite increased availability of knowledge for patients, asymmetries of power between patients and doctors persist, and these are exacerbated by fact that, even in Europe, healthcare systems are increasingly market oriented.

The idea that having an adequate knowledge of one’s health condition can benefit patients has been affirmed in the past few decades. Many public health campaigns are built on the idea that the public should be informed about which behaviours can expose them to health risks and which can protect them. From AIDS prevention to cancer screening campaigns, and the return of the salience of vaccination campaigns, the dominant idea is that knowledge and awareness can save lives. If the “knowledge is power” saying is true, the internet can certainly be considered an empowering tool. When a larger portion of the public started to use the internet, researchers were quick to indicate the empowering potential of the access to information for patients. While this applies more to the Global North, the impact of the internet on health has more recently been significant also in the Global South.^2^ However, it was also quickly understood that there were limits to that empowerment because patients might not be interested in looking for information, might not have the digital literacy to do so or might not be willing or able to challenge doctors on medical issues.^3^

Since the early 2000s, the internet has been offering an increasing number of resources to obtain medical knowledge, to contact medical professionals and, even more so, to connect with other patients. Social media provide new spaces in which patients can exchange information, data and scientific articles and have allowed the development of forms of treatment activism. Social media also represent a platform that can make visible the problems encountered by patients. One example of such visibility is the hashtag #doctorsaredickheads. Since October 2018, this hashtag has been used thousands of times on Twitter to discuss misadventures, cases of malpractice and episodes of coldness or lack of empathy that patients have encountered. This hashtag and the discussions it has generated are an important reminder of the fact that even today, when medical information has never been easier to access, the relations between patients and medical professionals are still complex and difficult.

The encounters between patients and doctors can be considered “micro-political situations that reflect and support broader social relations, including social class and political-economic power,” as the sociologist and medical doctor Howard Waitzkin wrote.^4^ The stories presented in the tweets using the hashtag showed how women in particular were more vulnerable during encounters with the medical system. Following the feminist slogan “The private is political,” I consider medical consultation to be a private moment that shows tensions linked to gender, but also race, and class—inequalities that define society outside the medical cabinet. These tensions are as important as the scientific and medical notions that drive the doctor–patient relationship. Indeed, biomedicine itself is not foreign to political tensions.

## Women, Breast Cancer and Biomedicine

“I am a doctor, dear, and I know.”^5^

“The Yellow Wall-Paper”

Charlotte Perkins Stetson

The female body is caught in the grips of pathologisation and naturalisation. Pain, particularly pain related to the reproductive functions, is considered a natural aspect of women’s lives, and this makes it harder to diagnose several diseases, such as endometriosis. At the same time, physiological processes such as menopause are constructed as a disease in need of a cure. Many physical problems are hurriedly attributed to anxiety or depression, shifting the attention from the body to the mind and thus hindering women’s access to the kind of treatment that could relieve their problems. Aesthetic standards that dominate the life of women can creep into the medical office, and characteristics such as weight are pathologised even when they are not directly affecting women’s health. The history of medicine and the social sciences of medicine have also shown how, from diethylstilbestrol to the Dalkon Shield intrauterine device and silicone breast implants, several drugs and medical implants have been damaging and even fatal for women.^6^

Breast cancer is an interesting lens through which to explore the tensions between patients and medical professionals. The disease touches a part of the body considered to be a symbol of femininity, and several gendered biases are condensed in its history and public representation. My research into this disease revealed that the relationship between women and the medical establishment continues to be difficult and sometimes even abusive. Between 2012 and 2014, I conducted fieldwork in France and Italy, where I explored the impact of surgical therapies for breast cancer—mastectomy, conservative surgery, and breast reconstruction. Between 2017 and 2018, I explored the experience of patients living with metastatic breast cancer in the United Kingdom. During these years I conducted more than 140 interviews with patients and with medical professionals and breast cancer activists.

Despite the therapeutic progress, breast cancer is still one of the cancer subtypes with a high incidence and mortality in Europe. In the past decades, the disease has received increased visibility and fuelled numerous cause-related marketing campaigns. October is internationally recognised as Breast Cancer Awareness Month, during which products are painted pink and their purchase is presented as a way to support the search for a cure. While the pink ribbon movement originated in the United States, it has spread globally. The message of the awareness campaigns focuses on individual strategies of prevention and early diagnosis. Metastatic breast cancer, the terminal stage of the disease in which cancer has spread outside the breast, is absent or very scarcely mentioned.

Although progress in the treatments of metastatic breast cancer has improved the prognosis for patients in the past decades, epidemiological studies show that survival time is still around three years. However, information and awareness campaigns for breast cancer do not tackle these issues and instead deliver a simplified message based on the assumption that healthy lifestyles—diet, physical activity and the elimination of alcohol and smoking—can significantly reduce the risk of developing breast cancer and that, if there is a cancer, it is essential to diagnose it as soon as possible.^7^ Lifestyle and early diagnosis have a limited effect in influencing the prognosis and survival, but these kinds of messages that invite women to act to protect their health are extremely convincing. Many women try to put such advice into practice by controlling their lifestyle and trying to access screening. However, the women I met were not protected by their knowledge and they still had to face conflict and malpractice.

## You Are So Overanxious: Breast Cancer Patients and the Difficulties of Being Heard

Despite the emphasis that breast cancer awareness campaigns have put on early diagnosis in the past few decades, many of the patients who participated in my research had difficulties accessing screening.^8^ The story of Mary, an English patient in her 50s, quite effectively captures these experiences. ^9^ Years before the interview she consulted her general practitioner (GP) because she felt a “bumpy area” in her breast. The doctor did not consider the problem significant and attributed Mary’s worries to anxiety. Mary was relieved at first, but because the problem persisted, she went back to the GP:

But six months later I thought “this bumpy area is still here.” So I went back to the GP, a different GP, and he said “I agree with my colleague [that you shouldn’t be referred] but you are so overanxious and I’ll send you [for a mammogram] just to put your mind at rest.” So, I went to do this mammogram, and I was diagnosed with primary breast cancer. So [this] delayed the diagnosis for six months.

This kind of experience shows the complexity of the doctor–patient relationship. Mary was aware that something was wrong, but she welcomed the doctor’s reassurance as a relief. This was not due to a passive or deferential attitude on Mary’s behalf but to the social power of the medical opinion. Doctors are the custodians of truth on the disease and on the body, so Mary’s embodied knowledge was dismissed as anxiety. Mary, like other women in her position, was brought up to trust the professional opinion. However, Mary’s embodied knowledge pushed her to insist for a referral that brought her to a cancer diagnosis. Later in the interview, Mary told me how she started a formal complaint against the doctor who caused a delay in her diagnosis, but she unfortunately encountered other difficulties. Several years after the early breast cancer diagnosis, Mary was diagnosed with metastatic breast cancer, and in this case her encounter with the medical system was also disappointing:

I have had back pain for a long time, but this became something else. I went to the GP and he gave me a muscle relaxer. Again a useless [GP] because there’s a GP with all my records knowing that I had a delayed diagnosis [of breast cancer], I go with my back pain and … he gave me a muscle relaxer, twice I visited him … so my lack of faith is at the GP level, is where I feel let down, where ignorance has been shown.

Many patients acquired an in-depth knowledge of their disease, of the available therapies, of the risks of relapse and of the ways in which the latter can manifest itself. Mary was aware that strong pain should be checked as a possible sign of a relapse in a patient with a previous breast cancer diagnosis. This is a well-known clinical practice, and it is probably this to which Mary referred when talking about “ignorance shown.” It would be reassuring to discount these cases as bad luck, but they were frequent and had in common the incapacity to recognise the relevance of illness experiences and embodied knowledge among women patients.

This was also the case of Amber, a woman in her 60s who, like Mary, had a previous breast cancer diagnosis and went back to see her GP in 2016 because she was not feeling well. The GP referred her for an ultrasound scan to the liver, but the scan was to be conducted in a gastroenterology department rather than in an oncology department. Still, the department was aware of Amber’s clinical history because she had informed them herself.

They wrote it down in their notes [the information concerning the previous breast cancer diagnosis] and I assumed they would realize that meant that I thought that it was coming back. In fact I told them that that was what I thought but they didn’t take any note [of my thoughts] and I didn’t realize that they didn’t put two and two together. I assumed that any gastroenterologist consultant looking at a liver that has possible metastasis would realize that what I was saying made sense. It didn’t occur to me that they wouldn’t get that, and I was given a liver biopsy that they proceeded to test for everything under the sun and they eventually discovered that it was breast cancer.

As in Mary’s case, for Amber the main problem was not the lack of information on the part of the patient but that the medical personnel did not pay attention to the suggestions and the knowledge that she shared with them. They dismissed it, thus causing a delay in Amber’s diagnosis. Like Mary, Amber also filed a complaint. Both women said that this decision was in part motivated by the fact that they hoped that this could change the behaviours of medical professionals and could spare other women the same difficulties they had.

## Making a Fuss for a Prosthesis: Encounters With Breast Surgeons

Patients’ autonomy and ability to make decisions did not increase when dealing with an elective intervention, such as postmastectomy reconstruction. Undergoing—or not undergoing—this kind of intervention did not affect the survival of the patients, but for many of them it was an important passage to regain a physical and psychological balance that the illness had compromised.

Different surgical techniques are available, with the simplest involving silicone gel prostheses, and the more complicated involves autologous transplants from the back or the belly of the patient. The interviews I conducted show complex relations between surgical techniques and the embodied experiences. Some women wanted to avoid an external object, such as a prosthesis. Others went against their doctors’ opinions, refusing interventions, such as the symmetrisation of the healthy breast or the reconstruction of the nipple, as they were interested only in obtaining a volume and avoiding wearing an external prosthesis. In some cases, the patients collected detailed information in preparation for the surgical consultation.

Denise, a French patient, conducted research online before talking to her surgeon about the possibility of having reconstruction with an autologous transplant. The doctor, however, reacted hostilely, answering, “You are not at the Galeries Lafayette [a famous Parisian department store]! It’s not you who should decide.” Conflictual situations like this, which were painful for the patients, were linked to the fact that many surgeons saw reconstruction as purely technical and considered the choice of the technique as their domain and not that of the patients. Dr. Claudine, a French surgeon, told me, “I am the professional. I have 15 years of experience” and “People are unfortunately very distorted by the media and by internet. They think they know it all with *Santé Magazine* and company.” She emphasized that her years of study and experience qualified her to choose for the patient and explained how the information that patients obtained was unreliable.

The medical authority invoked to choose the best technique for the patient was the same that in some cases made possible the underestimating the symptoms of the women that received the adulterated *Poly Implant Prothése* (PIP) breast implants. Jacqueline, for example, was a French patient who had to have her implant removed because of the adulteration and was involved in an association of the victims of the fraud. She told me the following during an interview:

In the beginning, they told to all these women [who had the PIP implants] that they were hysterical and that they were crazy, but gently crazy, eh, not nastily crazy, not psychiatrically crazy, but still crazy. And, if you want, women like me, who had a breast cancer, for the population, but even for many doctors, we were really lucky to have recovered. I have recovered. Why should I make a fuss for a prosthesis?

The PIP prostheses scandal affected many women in France and beyond who had received an implant after a mastectomy or for aesthetic reasons. Several years passed before it was understood that the high rupture and accident rates of this brand of implants were too high, and before the factory was inspected showing that the owner was using industrial grade rather than medical silicone.^10^ In the preceding passage, Jacqueline shows how difficult it is for women to be taken seriously by both the medical system and society at large.

Women are often treated with paternalism and superficiality and defined, as Jacqueline aptly suggested, as “gently crazy.” This definition is similar to the references to overanxiety that were used with Mary, while the preference for a specific surgical technique in Denise’s case was dismissed with a reference to shopping (“You are not at the Galeries Lafayette”)—an activity considered feminine and futile. Many of the women I met had to deal not only with cancer but also with the indifference of members of the medical profession who did not take their experience into account or underestimated their symptoms, or both.

Mary, Amber, Jacqueline and Denise, like other women I have met in past years in different European countries, were middle class, with good jobs and degrees and were generally well positioned to access detailed and reliable medical knowledge. This did not protect them from conflicts with the medical establishment. On the contrary, these conflicts were often exacerbated by the information held by the patients. Many patients thought that their experience and knowledge would help them to have a more active role in their therapies, but in the current organisation of the doctor–patient relationship, this was not possible.

## Knowledge Without Power: Women and the Healthcare System

The problems that patients had with the medical establishment were mostly interpreted as individual difficulties deriving from a lack of empathy on the doctor’s part. In Twitter debates such as the one around #doctor-saredickheads, several doctors affirmed that medical professionals should learn to listen and pay attention to patients’ needs. These desirable changes certainly could improve the experience of many patients. However, limiting the analysis of the problem to empathy risks obscuring other dimensions.

In many cases, the doctors with whom the patients I had met clashed had a gatekeeping function and decided whether the patients would have had access to other medical professionals, to further screening, or to more complex surgical techniques in the case of reconstruction. In the United Kingdom, GPs have to decide when to refer a patient for further testing or treatments and are therefore in a key position because their decisions significantly influence both the well-being of the patient and the financial equilibrium of the medical system.

They are, furthermore, implicitly and explicitly asked to pursue the containment of costs to the detriment of the quality of health care.

Research has shown how gatekeeper systems in healthcare are associated with lower survival times for patients with cancer diagnoses.^11^ In the United Kingdom, the gatekeeping process was accelerated in the 2010s with new market-based healthcare reforms that have increased the pressure on GPs to keep under control the level of health-care expenditure.^12^ A managerial system that limits not only the patients’ capacity for action but also that of the medical professionals is obviously not the ideal kind of context for receptivity and support for patients’ needs.

In the same way, for women who wish to undergo a specific type of breast reconstruction, surgeons are the gatekeepers of newer techniques and can omit informing the patients or oppose requests that they find too technical and detailed and that involve skills that only a limited number of surgeons have, such as microsurgery. I observed, for example, that the wish to defend professional prestige and the wish not to lose patients very likely influenced the behaviour of several reconstructive surgeons in France. In the context of an elective operation, such as breast reconstruction, many patients see their preferences diminished by the invocation of technical competence, presented as the domain of the doctors. Old stereotypes and gender inequalities can be adapted to the needs of increasingly market-based healthcare systems. In such a context, class inequalities are added to gender inequalities. A limited number of affluent patients can have access to the luxury private sector. For many other patients, this is not possible, or it is only in part. In healthcare systems that allow it, such as the French system, this often lead patients to mix treatment in the private and public sectors.

The patients’ experiences that I briefly described in this article invite us to analyse the tensions between patients and doctors within the processes of the privatisation of public healthcare systems and to resist simplistic solutions such as calling for greater information among patients or greater empathy on the doctors’ part, because these changes, although very welcome and important, do not represent a solution to remove the obstacles to access quality healthcare.

## Notes

1 The data presented here are based on two research projects, one funded by the Can-céropôle Île-de-France and another funded by a Newton International Fellowship of the British Academy. The article was written while I was a Wellcome Trust Fellow in Humanities and Social Science, grant number 212736/Z/18/Z.

2 For an example of a critical analysis, see Marine Al Dahdah, “Health at Her Fingertips: Development, Gender and Empowering Mobile Technologies,” *Gender, Technology and Development* 21, no. 1–2 (2017): 135–51.

3 Michael Hardey, “Doctor in the House: The Internet as a Source of Lay Health Knowledge and the Challenge to Expertise,” *Sociology of Health & Illness* 21, no. 6 (1999): 820–35, is an example of the optimistic approach; Flis Henwood et al., “‘Ignorance Is Bliss Sometimes’: Constraints on the Emergence of the ‘Informed Patient’ in the Changing Landscapes of Health Information,” *Sociology of Health & Illness* 25, no. 6 (2003): 589–607, is one of the first works to point out the limits of patients’ empowerment through the internet.

4 Howard Waitzkin, *The Politics of Medical Encounters: How Patients and Doctors Deal with Social Problems* (New Haven, CT: Yale University Press, 1991), 9.

5 Charlotte Perkins Stetson, “The Yellow WallPaper,” *The New England Magazine,* January 1892, 652.

6 See Susan E. Bell, *DES Daughters: Embodied Knowledge and the Transformation of Women’s Health Politics* (Philadelphia, PA: Temple University Press, 2009); Chikako Takeshita, *The Global Biopolitics of the IUD: How Science Constructs Contraceptive Users and Women’s Bodies* (Cambridge, MA: MIT Press, 2012); and Cinzia Greco, “The *Poly Implant Prothèse* Breast Prostheses Scandal: Embodied Risk and Social Suffering,” *Social Science & Medicine* 147 (December 2015): 150–57.

7 For an overview of the issues of breast cancer campaigns, see Gayle Sulik, *Pink Ribbon Blues: How Breast Cancer Culture Undermines Women’s Health* (Oxford, UK: Oxford University Press, 2012).

8 Breast cancer is one of the cancers for which screening has been most promoted, and the healthcare systems with the most developed screening programs might have a problem of overdiagnosis (i.e., even tumours that would have never become dangerous might be diagnosed and treated). At the same time, the literature on breast cancer screening is sometimes critical of self-initiated mammograms, as it considers it more efficient to have mammograms at fixed intervals for all the women targeted by screening; see, for example, Samiratou Ouédraogou et al., “Breast Cancer Screening Programmes: Challenging the Coexistence With Opportunistic Mammography,” *Patient Education and Counseling* 97, no. 3 (2014): 410–17. The risk of such an approach is that women who identify symptoms but are not in the age range targeted by screening, or have rapidly developing cancers, might find it difficult to obtain a mammogram.

9 All the names used are pseudonyms.

10 Greco, *“Poly Implant Prothèse,*” 150–57.

11 Peter Vedsted and Frede Olesen, “Are the Serious Problems in Cancer Survival Partly Rooted in Gatekeeper Principles? An Ecologic Study,” *British Journal of General Practice* 61, no. 589 (2011): e508–12.

12 Clare Gerada, “From Patient Advocate to Gatekeeper: Understanding the Effects of the NHS Reforms,” *British Journal of General Practice* 61, no. 592 (2011): 655–56.

